# Remote Memory in Epilepsy: Assessment, Impairment, and Implications Regarding Hippocampal Function

**DOI:** 10.3389/fneur.2022.855332

**Published:** 2022-04-08

**Authors:** Sanya Rastogi, Kimford J. Meador, William B. Barr, Orrin Devinsky, Beth A. Leeman-Markowski

**Affiliations:** ^1^Epilepsy and Cognition Laboratory, Veterans Affairs, New York Harbor Healthcare System, Research Service, New York, NY, United States; ^2^College of Arts and Science, New York University, New York, NY, United States; ^3^Department of Neurology and Neurological Sciences, Comprehensive Epilepsy Program, Stanford University School of Medicine, Palo Alto, CA, United States; ^4^Department of Neurology, Comprehensive Epilepsy Center, New York University Langone Health, New York, NY, United States; ^5^Department of Neurosurgery, New York University Langone Health, New York, NY, United States; ^6^Department of Psychiatry, New York University Langone Health, New York, NY, United States

**Keywords:** remote memory, long-term memory, consolidation, autobiographical memory, episodic memory, epilepsy, seizures, hippocampus

## Abstract

Studies of epilepsy patients provide insight into the neuroscience of human memory. Patients with remote memory deficits may learn new information but have difficulty recalling events from years past. The processes underlying remote memory impairment are unclear and likely result from the interaction of multiple factors, including hippocampal dysfunction. The hippocampus likely has a continued role in remote semantic and episodic memory storage over time, and patients with mesial temporal lobe epilepsy (TLE) are at particular risk for deficits. Studies have focused on lateralization of remote memory, often with greater impairment in left TLE, which may relate to verbal task demands. Remote memory testing is restricted by methodological limitations. As a result, deficits have been difficult to measure. This review of remote memory focuses on evidence for its underlying neurobiology, theoretical implications for hippocampal function, and methodological difficulties that complicate testing in epilepsy patients.

## Introduction

Anterograde memory deficits reflect the inability to form new memories, while retrograde amnesia refers to the loss of prior learned information. These forms of memory dysfunction provide insight into the processes of memory formation, consolidation, and retrieval. Nearly 50% of people with epilepsy have anterograde and retrograde memory impairments (1) which are often multifactorial in etiology, due to underlying hippocampal pathology (2, 3), frequent seizures (4, 5), early age of onset (6, 7), and long epilepsy duration (8). In 1953, patient H.M. developed severe anterograde and moderate, temporally graded retrograde amnesia following bilateral mesial temporal lobe resection for refractory epilepsy (9–11). H.M. revolutionized our understanding of the hippocampus' role in memory formation and paved the way for studies of anterograde memory in epilepsy. Remote retrograde memory deficits in epilepsy patients, however, are not well-understood. Patients with remote memory deficits may learn new information but have difficulty recalling events from long ago (12). The time point at which a memory is considered remote is ill-defined, but is typically ≥1 year (13). Patients with mesial temporal lobe epilepsy (TLE) are at particular risk for remote memory dysfunction (14).

Research in remote memory has focused on autobiographical and general semantic memory. Episodic autobiographical memory is the recollection of personal events (i.e., celebrating your 10th birthday), while semantic autobiographical memory refers to factual knowledge of your past (i.e., the school you attended). In contrast, general semantic memory is minimally influenced by personal experience. General semantic memory pertains to common knowledge of public figures and events, such as recognition of famous faces and awareness of news items.

## Anatomy of Remote Memory

The hippocampus and medial prefrontal cortex are activated during functional magnetic resonance imaging (fMRI) studies of remote memory. The anterior hippocampus is engaged during autobiographical recall and event construction, while the posterior hippocampus is implicated in event elaboration and spatial memory (15, 16). The hippocampus is comprised of multiple subregions that support establishment of new memories (“encoding”). Initial input from the entorhinal cortex (EC) projects *via* the perforant pathway to the *Cornu Ammonis* (CA), specifically area CA3, where intrinsic recurrent connections form a memory trace (17). The projection from the EC to CA3 may occur directly, or indirectly *via* the dentate gyrus, which reduces interference between similar information (17). The EC and CA3 activate CA1, which outputs information back to the EC and subiculum (17). Finally, the EC and subiculum relay information to neocortical locations (17). There is conflicting evidence, however, regarding the duration of the hippocampus' role in memory retrieval, resulting in opposing memory consolidation theories.

Across species, impairment of recent memory with relative preservation of remote memory is evident in the setting of hippocampal damage, a pattern termed “temporally graded retrograde amnesia” (18, 19). In contrast, patients with damage to lateral and anterior temporal neocortex can be amnestic for remote events (20). Autobiographical memory, for example, is intact in patients with damage restricted to medial temporal structures but is impaired when the injury extends to neocortex (21). These findings suggest that the hippocampus becomes less important for declarative memory storage and retrieval over time, the basic premise of the “standard model of systems consolidation” (SMSC) (22). This model proposes that new memories are encoded in both cortical and hippocampal regions, but are stored in progressively strengthened cortico-cortical synapses, while the hippocampal trace fades with time. The hippocampus is no longer required for retrieval once neocortical storage is sufficiently established (23). The hippocampal-dependent stage lasts up to 1 week after encoding, while the new information is gradually integrated into pre-existing cortical networks (24). Recall further strengthens cortico-cortical connections, resulting in the full transfer of the memory from the hippocampus to the neocortex (“consolidation”) (24). Retrieval of consolidated memories is independent of the hippocampus, predicting intact remote memory in patients with hippocampal lesions.

In contrast, the multiple trace theory (MTT) posits that spatial and context-specific details of an episodic memory are encoded in the hippocampus and remain stored there indefinitely, although some information is transferred to the neocortex (25). The entire hippocampal-neocortical pathway forms the consolidated memory trace. During each subsequent retrieval, the trace is reactivated, and a new, slightly different trace is established, so that over time, multiple related traces exist to facilitate retrieval. The MTT also emphasizes a distinction between episodic and semantic memory, in that both rely on the hippocampus for encoding, but general semantic memory eventually becomes independent of the hippocampus. While hippocampal traces contain contextual, spatial, and temporal details, cortical traces are semantic and context-free. Personally significant semantic memories, however, may have continued mesial temporal involvement.

The MTT predicts temporally graded retrograde amnesia for general semantic memory following hippocampal injury (26). Hippocampal damage would preferentially affect recent general semantic memories, as remote memories would be well-established in the neocortex. Yet, numerous rodent studies provide opposing evidence, with flat retrograde amnesia gradients for semantic memory after complete hippocampal damage (26–29). The MTT also posits that episodic memories become more strongly established in the hippocampus over time as the number of traces pertaining to that memory increases. Recent episodic memories, which have fewer memory traces, should be more disrupted by partial hippocampal lesions than remote memories (30). While the MTT places importance on the amount of damaged tissue, the specific structures affected may be the relevant factor. Several rodent studies, however, showed that remote memories were more vulnerable to partial hippocampal damage than recent memories, even when the precise location of hippocampal damage varied (27–29). Further, electrophysiologic unit recordings (31, 32) and calcium imaging (33, 34) in rodents showed a constant or decreasing number of place cells representing spatial memories, which contrasts the MTT's prediction that the number of activated cells increases when re-experiencing a specific context. Although much of the rodent literature supports the MTT, conflicting evidence surrounding the memory consolidation process remains.

Derived from the MTT, the trace transformation theory (TTT) (35) accounts for changes in memories over time, transforming from highly detailed to coarse representations. The TTT emphasizes the role of the posterior hippocampus and its connections to perceptual posterior neocortical regions in supporting finely detailed memories and the anterior hippocampus for remembering the overall “gist.” The anterior hippocampus connects with anterior neocortical regions, notably the medial prefrontal cortex, where schemas represent common features across multiple events. These pathways are reactivated over the memory's lifetime, shifting to and from finely or coarsely detailed recollections. While detailed recollection will always require hippocampal activation, schematic memory may be supported by the neocortex alone.

The MTT and TTT are supported by neurophysiological data from patients with medically refractory epilepsy. Intracranial EEG recordings demonstrated sharp wave ripples, oscillatory patterns supporting memory reactivation, in the hippocampi during recall of autobiographical memories (36). Ripple rate was higher during retrieval of remote memories compared to more recent events, supporting a continued role of the hippocampus. Ripples preferentially occurred in the anterior hippocampus, which the authors considered “compatible with [a] gist-like recollection strategy.” With increasingly remote memories, ripple rate patterns associated with autobiographical events gradually became more similar to patterns of semantic retrieval, consistent with the theory that memory traces evolve over time to more general representations. Further, ripples correlated with cortical high frequency band activation, including posterior neocortex and medial prefrontal cortex, supporting the theory of recurrent hippocampal-neocortical interplay in these regions during autobiographical recall. Electrocorticographic data is consistent with functional MRI activation of hippocampal, medial frontal, and parietal regions during recall of remote autobiographical events (37).

## Remote Memory Deficits in Epilepsy

Remote memory abilities in epilepsy patients could also help distinguish between these models. If the SMSC is true, remote memory should be relatively unaffected in patients with mesial TLE, although patients with neocortical epilepsy may be impaired. If the MTT/TTT is correct, patients with either mesial TLE or neocortical epilepsy may have impaired remote memory. The distinction may be blurred, however, in that focal epilepsy negatively impacts wide networks, such that mesial TLE may impair neocortical function. Further, the epileptogenic neocortical regions may lie outside of those supporting consolidation, such that remote memory could remain intact. Studies are limited by the inclusion of post-surgical epilepsy patients, in whom mesial and lateral structures are removed, and mixed TLE groups with either mesial or neocortical epileptogenic zones. Nevertheless, most studies demonstrated remote memory impairments in TLE (Table 1).

**Table 1:** Remote memory deficits in epilepsy patients.

**Study**	**Subjects**	**Type of remote memory tested**	**Retrograde memory tasks**	**Findings**
Barr et al. (38)	Post-surgical: 6 LTL 6 RTL 6 Healthy controls	Autobiographical Public/semantic	Famous Faces Test (39, 40) Television Test (41) Goldberg-Barnett Remote Memory Battery (42)	LTL < RTL = HC
Bergin et al. (12)	Pre-surgical: 14 LTLE 19 RTLE 18 Left Ex-TLE 15 Right Ex-TLE 10 PGE 30 Healthy Controls	Public/semantic	Questionnaire assessing knowledge of public events	TLE < Ex-TLE = PGE = HC RTLE = LTLE R-ExTLE = L-ExTLE
Lah et al. (43)	Post-surgical: 15 LTL 15 RTL 15 Healthy Controls	Autobiographical Public/semantic	Australian Remote Memory Battery (ARMB)— Famous Faces and Famous Events (44) Public Fluency Test (PFT)— Famous Names and Famous Events (45) Autobiographical Fluency Test (AFT)— Names and Events (45)	Famous events: RTL < HC = LTL Famous names: LTL < RTL = HC AFT-Names: RTL < HC = LTL AFT-Events: LTL = RTL = HC
Lah et al. (46)	Pre-surgical: 15 LTLE 14 RTLE 15 Healthy controls	Autobiographical public/semantic	Australian Remote Memory Battery (ARMB)—Famous Faces and Famous Events (44) Public Fluency Test (PFT)—Famous Names and Famous Events (45) Autobiographical Fluency Test (AFT)— Names and Events (45)	Semantic Memory: LTLE < RTLE = HC Autobiographical: TLE < HC
Viskontas et al. (47)	Pre-surgical: 8 LTLE 5 RTLE Post-surgical: 6 LTL 6 RTL 22 Healthy Controls	Autobiographical	Autobiographical Memory Interview (48)	Personal episodic memory: TL < HC = TLE Personal semantic memory: TLE = TL = HC
Voltzenlogel et al. (49)	Pre-surgical: 19 LTLE 19 RTLE 35 Healthy controls	Autobiographical Public/semantic	Autobiographical Memory Interview (48) Modified Crovitz Test (50, 51) Public Events Test (52) Famous Scenes Test (53) Famous Faces Test (Denkova and Manning, unpublished) Dead/Alive Test (54)	LTLE < RTLE < HC

*Ex-TLE, extra-temporal lobe epilepsy; HC, healthy controls; L, left; LTL, left temporal lobectomy; LTLE, left temporal lobe epilepsy; PGE, primary generalized epilepsy; R, right; RTL, right temporal lobectomy; RTLE, right temporal lobe epilepsy; TL, temporal lobectomy; TLE, temporal lobe epilepsy*.

### Autobiographical Memory in Epilepsy

Autobiographical memory was impaired in TLE patients compared to healthy controls, although it did not correlate with subjective memory complaints (55), which deserves further study. The Autobiographical Memory Interview (AMI) and Modified Crovitz Test were intact for personal semantics in pre-surgical TLE patients, the majority with mesial lesions, but memory for autobiographical episodes was impaired relative to controls (49). Similarly, subjects with mesial TLE, a subset post-surgical excision with lateral resections of unclear extent, had intact semantic autobiographical memory, but poorer episodic memory on the AMI than controls across all time periods (47). Pre-surgical TLE patients, the majority with hippocampal pathology, were impaired when generating friends' names (semantic autobiographical memory) on the Autobiographical Fluency Test, with the right TLE patients performing worse than controls when generating autobiographical events (46). Pre-surgical data largely support the MTT, in that hippocampal lesions were associated with impaired episodic remote memory. Data regarding semantic autobiographical memory, however, lend inconsistent support for the hypothesis that semantic memory is consolidated strictly in neocortex (46).

Variable autobiographical memory performance is evident post-anterior temporal lobectomy (ATL). Patients post-ATL had normal autobiographical event recall on the Autobiographical Fluency Test (43). In contrast, a small sample of post-left temporal lobectomy patients had impaired autobiographical memory for pre-surgical events (38). Pre- and post-surgical patients also had impaired memory for autobiographical events, in that memories contained impoverished perceptual detail (56). Reasons for variable results are unclear, but may relate to the laterality or extent of resection. One study included patients post-ATL, which removes neocortical and mesial structures, and patients post-selective amygdalohippocampectomy (SAH), which spares neocortex (56), but did not directly compare the two groups.

### General Semantic Memory in Epilepsy

General semantic memory is consistently impaired in TLE, pre- and post-temporal resection. Like studies of autobiographical memory, data support the MTT/TTT, demonstrating a continual role for the hippocampus in retrieval of remote memory, but do not suggest that consolidated semantic memories are restricted to neocortex. Hippocampal ripples, for example, were of similar magnitude during general semantic and autobiographical episodic remote memory retrieval (36). Patients with TLE with either mesial or neocortical lesions had poorer recall and recognition for general semantic memory of public news items compared to healthy controls, extratemporal epilepsy patients, and primary generalized epilepsy patients (12). Pre-surgical left TLE patients, most with hippocampal pathology, had impaired recall and recognition of public knowledge, assessed by the Famous Faces, Famous Events, and Public Fluency Tests (46). Pre-surgical patients with TLE, most with mesial lesions, had poorer performance than healthy controls when naming famous faces and scenes, answering questions about news events, and indicating whether famous figures were dead or alive (49). Post-left temporal lobectomy, general semantic memory deficits were also evident when naming famous faces, providing knowledge about famous events and people, and recognizing names of short-lived television shows (38).

### Lateralization of Remote Memory in Epilepsy

Evidence for lateralization of remote memory in epilepsy patients is mixed. Some data indicate no relationship between laterality of the seizure onset zone and memory for public news items (12) or autobiographical memory (47). Both left and right TLE groups were impaired, for example, when generating friends' names (46). Other data suggest lateralization of certain types of remote memory, with impaired autobiographical event fluency in right TLE and deficits regarding public knowledge in left TLE (46). Reduced right hippocampal activation was evident in patients with right mesial TLE compared to controls during remote autobiographical event recall, not explained by hippocampal volume (37). In contrast, hippocampal ripples occurred preferentially in the left anterior hippocampus during autobiographical event recall, in patients with either left, right, or bilateral seizure onset zones (36). Overall, when an asymmetry was present, patients with left TLE were typically more impaired, as seen with tests of public knowledge (38, 46, 49) and autobiographical memory (38, 49). Whether these findings relate to the verbal nature of tasks is unknown. Object naming and semantic and phonological fluency correlated with general semantic and autobiographical memory, which could explain group differences (46), although not seen in all studies (12).

### Contributing Factors to Remote Memory Deficits

Several studies examined the effects of seizure-related variables on remote memory. Epilepsy duration and history of generalized seizures did not impact memory for autobiographical or public facts and events (46). Nor was autobiographical memory associated with hippocampal sclerosis or temporal lobe resection (47). Further, a history of status epilepticus did not predict remote memory for public events (12). Data regarding the impact of antiseizure medications (ASMs), however, are conflicting. Post-temporal lobectomy patients taking ASMs had poorer autobiographical semantic fluency than patients not taking ASMs (43), and pre-surgical TLE patients taking ASM polytherapy had impaired autobiographical event fluency compared to patients taking monotherapy (46). The number of ASMs also negatively correlated with recognition of famous events (46). No correlation was seen, however, between the number of ASMs and recall of news events (12), although studies differed in testing procedures, types of epilepsy included, and degree of seizure control. Data regarding age of onset are also variable. Poorer memory for famous events correlated with earlier age of onset (46), but this was not seen in other studies (12, 47, 49). Effects of seizure frequency are mixed, as well. Patients with less frequent seizures had better memory for personal (5) and public events (5, 12) than patients with more frequent seizures. Seizure-free patients also had greater autobiographical semantic fluency (for the previous 5-year period) than patients with active epilepsy (43). Other data demonstrated no significant correlation between seizure frequency and memory for personal events, public events, or famous faces (49). Results may be limited, however, by difficulty in measuring seizure frequency. Future studies should consider the potential impact of interictal epileptiform discharges and psychiatric comorbidities.

While similar deficits in autobiographical memory were seen in subjects with or without surgical resection (47, 56), the impact of surgery has not been studied within individual subjects or in general remote episodic or semantic memory. Further, current studies may be limited by a lack of detail regarding the extent of neocortical resection (47). “Standard” ATLs are not standardized, in that variable amounts of neocortical and mesial tissue may be resected. The impacts of surgical approach and size of the resection are unknown. Comparison of performance post-ATL with more selective surgeries may help clarify the role of temporal neocortex and duration of hippocampal involvement in remote memory. SAH, however, can disrupt cortical and subcortical fibers in the approach to mesial structures. Depending upon the surgical approach, SAH could be expected to impair remote memory function in a similar manner as ATL (57, 58). Future studies may consider the effects of laser induced thermal therapy (LITT) on remote memory, as this technique can create more selective lesions within the mesial temporal lobe. Stereotactic laser amygdalohippocampectomy can preserve anterograde verbal memory, highlighting the importance of extra-hippocampal structures in memory performance (59). Postoperative studies may also be enhanced by including MRI quantification of the resected structures as a covariate.

## Methodological Issues in Assessing Remote Memory

Studies of temporal lobe damage provide conflicting support for the SMSC, MTT, and TTT. To understand variability across studies, we must consider the tasks used (Table 2) (39, 40, 44, 45, 48, 50, 51, 54, 60, 61). Several methodological factors limit remote memory testing, including a lack of control over the time and circumstances of encoding. Long-standing anterograde memory deficits may result in poor initial memory acquisition, which may falsely appear as remote, retrograde deficits. Further, the date of the event may not correspond to the time of acquisition, such that the “age” of a memory may be uncertain. It is also difficult to control the frequency of memory rehearsal or re-exposure, which may allow easier recall or recognition. Tests of autobiographical fluency (45), for example, can include recurrent events.

**Table 2:** Remote memory tests.

**Test**	**Type of remote memory tested**	**Testing protocol**	**Time periods tested**	**Scoring**
Autobiographical Memory Interview (AMI) (48)	Autobiographical	(1) *Autobiographical Incidents Schedule*: describe specific memories from past; three per time period (2) *Personal Semantic Memory Schedule:* knowledge of facts about past; three items per time period	Childhood Early adult life Recent events (past 5 years)	*Autobiographical Incidents:* 27 pts total (9 pts per time period) *Personal Semantics:* 63 pts total (21 pts per time period)
Galton-Crovitz Test (50, 51)	Autobiographical	Recall life events related to each of 20 high-frequency nouns (e.g., “holiday,” “birthday”) and estimate date of memory produced	N/A	0–3 scale for each memory produced
Autobiographical Fluency Task (45)	Autobiographical	Recall of personal events and names of acquaintances from each time period in 90 s	Preschool Primary school Secondary school Five years post-school Current	Number of items reported per time period in 90 s
Famous Faces tests (39, 40, 44)	Public/semantic	Identify faces of famous people in photographs from six decades, categorized by decade and difficulty. For each target picture, two non-famous faces are used as foils, matched to targets by age, sex, and time period	N/A	Number or percent of faces recognized
Famous News Events tests (44)	Public/semantic	Questions relating to prominent events categorized by decade	N/A	Number or percent of events recalled/recognized
Dead or Alive Test (54)	Public/semantic	Recognize faces of alive vs. dead famous individuals State year and cause (natural or unnatural) of death.	N/A	Number or percent correct
Transient News Events Test (60, 61)	Public/semantic	Recall and recognition questions related to transiently popular news events, categorized by hemi-decade	Events since subject was 10 years old, from 1961 to 2013 (updated version until 2016)	Number correct

*NA, not applicable*.

Autobiographical memory is often complicated by emotional and contextual factors (62). Emotional events create vivid autobiographical episodic memories, often resulting in highly detailed “flashbulb” memories of the moment an event occurred. These emotionally-laden memories are more often rehearsed and more easily recalled, which could lead to an overestimation of remote memory abilities. Emotional memories also involve extratemporal regions, including frontal cortex, such that effects of TLE localization and lateralization are not straightforward. Third-party validation of self-reported memories may present challenges, as it may be difficult to find someone to corroborate the subject's responses, and the third party may not accurately remember events. It is difficult to establish response accuracy, and the detail required for a “correct” response is unclear.

Assessments of general semantic knowledge also pose several methodological issues. People who lived outside of the country in which the event occurred may not have been exposed to the material being tested. Exposure to news items may also vary based on interest (63). Further, questions regarding “recent” events will become outdated over time, requiring questionnaires to be updated frequently. Many tests assess material from the past several decades, of which younger subjects may be unaware. Related events or events of a similar nature may be easily confused. Lastly, some news items may be more prominent than others, appear in the news for greater lengths of time, or become popular again after several years, such that saliency may vary.

The Transient News Events Test (TNET) accounts for several of these methodological issues. It controls for saliency by using news items with a similar frequency of reporting, measured by the number of mentions in the New York Times Article Archive. Frequency of rehearsal is largely controlled by including events that had similar declines in the number of mentions over a 5-year period, allowing assessment of memories from restricted time periods. The variation in what can be considered a “correct” response is minimized, as the amount of detail required is clearly defined for each question. Finally, subjects are asked about events occurring when they were 10 years of age onwards, accounting for a possible lack of media exposure in early childhood.

Epilepsy poses additional challenges to remote memory testing. Early onset of epilepsy and resultant functional reorganization may have two consequences: anterograde and retrograde memory impairments may be difficult to differentiate, and different cognitive processes for encoding and retrieval may be used when compared to healthy subjects (64). Further, interrupted schooling may affect exposure to test items.

## Discussion

The literature lends insight into the brain regions involved in remote memory, though our current understanding of remote memory storage and retrieval remains limited. The exact role of the hippocampus is unclear, resulting in opposing theories of memory consolidation: the SMSC and MTT/TTT. The study of remote memory deficits in epilepsy can help to distinguish between these competing theories, as epilepsy may be associated with lesions of the underlying pathways. Data suggest a continued role for the hippocampus in remote general and autobiographical semantic and episodic memory and argue against the hypothesis that general semantic memory storage is restricted to neocortex. Studies are constrained, however, by methodological limitations of remote memory tests. We need improved, standardized testing for remote memory loss in epilepsy patients, and its inclusion in clinical neuropsychological batteries should be considered, as remote memory loss may be a source of disability not detected by current clinical testing. Further study of epilepsy-related factors and mechanisms of decline would help elucidate how seizures impair remote memory.

## Author Contributions

SR conducted the literature review and wrote the initial draft of the manuscript. KM contributed to the literature review and critical revision of the manuscript. WB and OD contributed to critical discussions regarding the topic. BL-M conceived of the manuscript and contributed to the literature review and drafts of the manuscript. All authors agree to be accountable for the content of the work.

## Funding

This work was supported by a New York University Dean's Undergraduate Research Fund (DURF) award (SR) and a United States (U.S.) Department of Veterans Affairs Clinical Sciences R&D (CSRD) Service Career Development Award (IK2 CX-001255) (BL-M).

## Conflict of Interest

The authors declare that the research was conducted in the absence of any commercial or financial relationships that could be construed as a potential conflict of interest.

## Publisher's Note

All claims expressed in this article are solely those of the authors and do not necessarily represent those of their affiliated organizations, or those of the publisher, the editors and the reviewers. Any product that may be evaluated in this article, or claim that may be made by its manufacturer, is not guaranteed or endorsed by the publisher.
